# Ligature of the Common Iliac Artery

**Published:** 1864-03

**Authors:** Daniel Brainard

**Affiliations:** Professor of Surgery in Rush Medical College


CHICAGO
MEDICAL JOURNAL.

VOL. XXI.] MARCH, 1864. [No. 3.

### ORIGINAL COMMUNICATIONS.

### LIGATURE OF THE COMMON ILIAC ARTERY.

By DANIEL BRAINARD, M. D.,

*Professor of Surgery in Rush Medical College.*

April 9th, 1863.—Called to visit Col. Scott, 19th Ills. Vols., who was wounded at the battle of Stone River. A musket ball had passed from before backwards through the thigh, entering below the pelvis and at the outside of the femoral artery, grazing the inside of the femur, and coming out of the buttock.

At the time of the accident, there was hœmorrhage, which was controled, as was supposed, by pressure on the femoral artery. The compression was continued about three weeks, during which time no hœmorrhage occurred. The wound suppurated and some small scales of bone came out at each orifice of the wound.

He was removed to his home in Chicago, and did well, although the wound remained open behind, until about the 5th of April, three weeks after the accident, when a small tumor formed in front, which was opened. A day or two after, a hœmorrhage took place from both openings. It was on account of this that my advice was asked. On the night of the 9th, at 11 o'clock, a copious hœmorrhage renewed, which was controled in a measure, but continued at intervals during the night.

10th.—Saw him at 10 o'clock, and applied the compressor over the femoral artery. This seemed to arrest the bleeding, but in about two hours it returned.

The bleeding had been so great as to threaten death, and I determined to tie the external iliac artery, not doubting from the history of the case that the hœmorrhage was from branches of the profunda femoris close to its origin.

With the aid of Prof. Freer and the Drs. Hurlburt, the ligature was placed upon the external iliac artery in the usual manner, as that described by Lisfranc; but on changing the position of the patient to remove the soiled bed clothing, the bleeding renewed as freely as ever. On a re-examination, the ligature was found to control the external iliac, and it was evident that the ischiatic artery was the one giving blood. The danger was urgent, and I enlarged the wound upward and outward, and placed a ligature on the common iliac artery. The anterior wound in the thigh was then enlarged, and a great quantity of coagula removed from it by the finger. No bleeding; patient under chloroform during the operation. Warm applications to the member; brandy and broth ordered.

11th, A. M.—Limb cool, but not cold; has been troubled with nausea and attempts to vomit, which gave pain in the wound; pulse 100, condition good. Ordered an enema and a solution of Soda Bi. Carb. with Gum Arabic for the vomiting. Broth continued.

12th.—Has considerable pain and tenderness in the region of the left kidney. Pulse 120; slept well during the night, with two doses of Acetum Opii; wounds commencing to suppurate.

13th.—Pulse 100; tenderness in left side diminished; takes broth with wine; slept well.

20th.—Cut of operation suppurates freely. Allowed beef broth and wine, with opiate at night.

24th.—Ligature on the external iliac artery came away.

May 1st.—Ligature on common iliac came away. Patient doing well.

May 12th.—Wound from operation healed.

From this time, he remained in good health until the early part of July, although the wound continued to suppurate and some small pieces of bone were discharged at the posterior orifice.

At this time he was attacked by a copious watery diarrhœa followed by typhoid fever, of which he died July 8th, three months after the operation.

